# Streamlined single shot safe harbor transgene integration in *C. elegans* using *unc-119* rescue.

**DOI:** 10.17912/micropub.biology.001230

**Published:** 2024-05-29

**Authors:** Katherine S. Yanagi, Nicolas Lehrbach

**Affiliations:** 1 Basic Sciences Division, Fred Hutch Cancer Center, Seattle, Washington, United States

## Abstract

Transgenic animals are an invaluable tool in model organism genetics. The ease of modifying the *C. elegans* genome through high-copy integration of transgenes facilitates the investigation of diverse and fundamental biological processes. However, generation of new multicopy integrated transgenes is limited by the time and labor cost. Further, many transgenes are integrated using non-specific DNA damaging agents. These DNA damaging agents cause unwanted mutations during the integration process and may have deleterious effects. A recently described method for CRISPR/Cas9-based integration of multicopy transgenes at safe harbor loci using Fluorescent Landmark Interference (FLInt) greatly increases the efficiency of multicopy transgene integration and mitigates issues related to off-target mutagenesis during integration. *unc-119* rescue is a simple and widely used phenotypic marker in *C. elegans* transgenesis and genome engineering. To streamline generation of multicopy transgenes via FLInt, we have generated a set of strains suitable for FLInt-mediated integration of transgenes using rescue of the *unc-119* mutant phenotype to select transgenic animals. We demonstrate the utility of this approach and outline a protocol that uses *unc-119* rescue as a selection marker for streamlined integration of multicopy transgenes at safe harbor loci.

**Figure 1. Single shot safe harbor transgene integration using unc-119 rescue:**
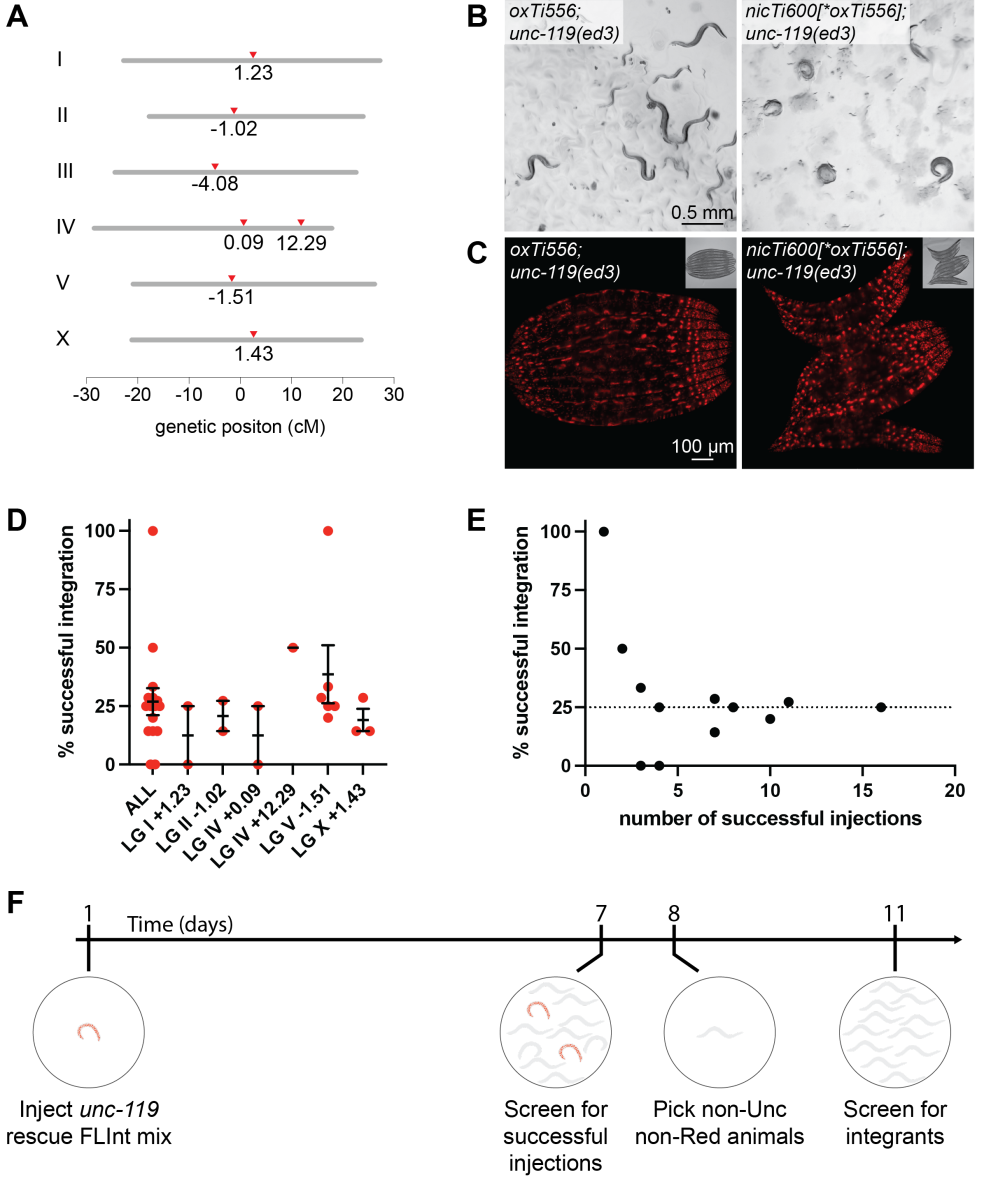
**(A)** Genomic locations of tdTomato landing pads edited to be suitable for FLInt with *unc-119* rescue [see also Table 1] **(B)** Bright field micrograph showing difference in posture and tracks (reflecting the *unc-119* mutant phenotype) caused by inactivation of the *Cbr-unc-119* rescue cassette in *oxTi556 .* Scale bar shows 0.5 mm. **(C)** Fluorescence micrograph showing expression of TdTomato is not altered by inactivation of the *Cbr-unc-119* rescue cassette in *oxTi556* . Scale bar shows 100 um. *nicTi600* was generated by CRISPR/Cas9 editing of the *Cbr-unc-119* resscue cassette in *oxTi556* . **(D)** Comparison of integration efficiency across the landing pad sites. The success rate for an integration event was calculated from the number of lines obtained from plates with successfully injected animals (plates with non-Unc). All integrations were carried out following the protocol described in the methods section. The number of successfully injected animals for each integration ranged from 2 to 16. **(E)** Comparison of integration efficiency as a function of number of successfully injected animals. All injections in which greater than 5 animals were successfully injected yielded at least one integrant. Injections with larger numbers of successfully injected animals consistently yield a 20-25% success rate. **(F)** Timeline for streamlined transgene integration using FLInt and *unc-119* rescue. 1-2 hours of hands-on work is required to carry out steps on days 1, 7, 8, and 11.

## Description

Integration of multicopy transgenes in *C. elegans* is a powerful tool widely used in studies of gene regulation and function (Nance & Frøkjær-Jensen, 2019). This technique is particularly useful in situations where stable and strong expression of a protein of interest or fluorescent reporter is required. Classical protocols for generation of multicopy integrated transgenes use DNA damaging agents to cause spontaneous chromosomal integration of extrachromosomal DNA arrays during repair of DNA damage (Evans, 2006; Mariol et al., 2013) . However, these approaches are time consuming and laborious, taking approximately two months and a large amount of manual screening to identify rare integration events. Undesirable outcomes of random transgene insertion using DNA damaging agents include mutagenesis of the genome, particularly at or near the integration site, and integration site-specific effects on transgenes’ pattern or level of expression. Transgene integration can be streamlined using optogenetically-induced DNA damage, though this approach does not mitigate issues caused by undesirable mutagenesis events that can accompany integration (Noma & Jin, 2018) . CRISPR/Cas9 technology has led to the development of improved methods for site-specific integration of multicopy transgenes at safe harbor loci that do not require use of non-specific DNA damaging agents (El Mouridi et al., 2022; Malaiwong et al., 2023; Nonet, 2021; Yoshina & Mitani, 2022; Yoshina et al., 2016) . Malaiwong and colleagues recently reported a method for CRISPR/Cas9-mediated integration of multicopy transgenes that uses mapped single-copy minimos transposon insertions as safe harbor loci for integration. This approach, Fluorescent Landmark Interference (FLInt), drastically reduces the time and labor costs of generating multicopy integrated transgenes and circumvents the major drawbacks of random integration using DNA damaging agents.

Genome engineering approaches including integration of multicopy transgenes can be streamlined by using simple phenotypic markers to identify transgenic animals. Rescue of the strong Unc phenotype caused by inactivation of the *unc-119* gene is widely used as a marker for many transgenesis and genome engineering applications in *C. elegans* (Maduro, 2015) . In FLInt, the mapped single-copy minimos insertions that serve as landing pads for transgene integrations typically contain a *Cbr-unc-119* rescue minigene, so it is not possible to use *unc-119* rescue as a phenotypic marker for transgene integration. To overcome this obstacle, we used CRISPR/Cas9 gene editing to inactivate the *Cbr-unc-119* rescue minigene found in several safe harbor loci suitable for FLInt (Fig 1A, Table 1). FLInt is more efficient at loci close to the center of chromosomes (Malaiwong et al., 2023) . As judged by expression of single-copy minimos transposon insertions, integration near the center of chromosomes is also likely to allow higher transgene expression than insertions into chromosome arms (Frøkjær-Jensen et al., 2016). We therefore inactivated *Cbr-unc-119* in at least one landing pad near the center of each chromosome. As expected, inactivation of the *Cbr-unc-119* minigene causes an Unc phenotype but does not alter expression of red fluorescent tdTomato from the safe harbor minimos insertion (Fig 1B, C). Thus, we can use *unc-119* rescue as a marker for transgenesis vis FLInt at the edited safe harbor loci.

We have used these strains to carry out FLInt-mediated integration using *unc-119* rescue as a marker for transgenesis. We have integrated a range of constructs into several of the edited safe harbor loci (Fig 1D). Approximately 25% of successfully injected animals yield a stable integration event, a similar integration efficiency to that previously reported for FLInt (Fig 1D, E) (Malaiwong et al., 2023) . A stably transmitted Unc-rescuing transgene (either a high-transmission array or integrant) provides a massive population-level growth advantage in a plate otherwise populated by Unc animals. Thus, plates that are likely to contain an integrant can be identified 7-10 days after injection as starved plates with a large population of non-Unc animals. Due to the efficiency of cutting at the tdTomato landing pad, we find that these animals almost invariably do not express tdTomato. Picking 8-10 single non-Unc animals from such plates is sufficient to (1) determine whether the plate contains a stable array or an integration event and (2) derive a homozygous line for the integration when present. Because *unc-119* rescued animals can be easily discriminated from non-rescued Unc animals on a dissecting microscope, the time taken for picking transgenic animals and phenotype scoring to identify homozygous integrations using this method is minimal ( Fig. 1F ).

Whilst carrying out integrations using this approach, we noted that the expression level of integrated transgenes can vary significantly between different lines. We did not determine the copy number of the integrated transgenes, but differences in copy number may explain variation observed. When integrating constructs that drive fluorescent protein under a constitutively active promoter, we have also noticed rare cases where an apparently integrated array shows mosaic or highly variable expression. We have also observed rare cases in which integrant lines show fertility defects. We also note that while we have not directly confirmed that transgene integration occurs at the predicted site, we have always observed the expected linkage in crosses carried out using the transgenic lines we generated. Taking these factors into account, it is ideal to verify that the construct is expressed at the desired level and in the expected anatomical pattern, and (if the integrated transgene is expected to lack toxic effects) ensure that the integration is not associated with defects in development or fertility. In practice, we find it is straightforward to isolate multiple independent integrated lines from a single round of injections (~10-20 animals successfully injected) and then select a line with the desired properties for downstream analysis. We include a protocol for streamlined FLInt using *unc-119* rescue (see Methods). In summary, we describe *unc-119* mutant safe harbor strains and a streamlined strategy that reduces that hands-on time required for highly efficient multicopy transgene integration using FLInt.

## Methods

*C. elegans* husbandry.

*C. elegans* were maintained on standard nematode growth media (NGM) at 20˚C-25˚C and fed *E. coli* OP50.

Microscopy.

Brightfield and tdTomato fluorescence images were collected on a Leica M165FC equipped with a Leica K5 sCMOS camera and using LAS X software. For fluorescence images, worms were immobilized for imaging using sodium azide and mounted on 2% agarose pads. For all fluorescence images, images shown within the same figure panel were collected using the same exposure time and were then processed identically.

CRISPR/Cas9 mediated inactivation of *Cbr-unc-119* in minimos insertions.

We used CRISPR/Cas9 and homology directed repair with a single stranded oligonucleotide (Ghanta & Mello, 2020) to inactivate the *C. briggsae unc-119* minigene in several mapped minimos lines (Frøkjær-Jensen et al., 2014) (see Table 1). To generate Cas9 RNPs targeting *Cbr-unc-119* , we mixed 30 pmol *S. pyogenes* Cas9 (IDT #1081058) with 95 pmol crRNA targeting the *C. briggsae* *unc-119* sequence CACCGAACAAGGAATCACAA (IDT custom guide RNA), and 90 pmol tracrRNA (IDT #1072532) in a total volume of 8.3 μl and incubated at 37°C for 15 minutes. We added 2.2 μg of an ssDNA oligo homology directed repair template designed to introduce a premature termination codon to the *Cbr-unc-119* minigene (with the following sequence: CCACCACCAAGCACCGAACAAGGAATCTGATCAAATCGGAGCTTGCGAAGAAAGCTCAAA), and 800 ng of pRF4 *rol-6* ( *su1006* ) plasmid as a co-injection marker. The final volume of the injection mix was adjusted to 20 μl with nuclease-free water. Following injection, desired edits were identified by the presence of Unc animals in the F2 generation.

Protocol for streamlined transgene integration using FLInt and *unc-119* rescue.

This protocol is a modified version of FLInt (Malaiwong et al., 2023) . We advise that users are familiar with the FLInt method before carrying out this protocol.

1. Prepare injection mixes.

1.1. Prepare RNP complexes (also see Ghanta and Mello 2020 for a detailed protocol for Cas9 RNP preparation), by combining the following:

· Cas9 (IDT #1081058), 0.5 μl of 10 μg/ml stock (30 pmol)

· tracrRNA (IDT #1072532), 5 μl of 0.4 µg/µL stock (90 pmol)

· tdTomato crRNA (targeting GGAGTTCAAGACCATCTACA), 2.8 µl of 0.4 µg/µl stock (95 pmol)

1.2. Incubate at 37˚C for 15 minutes to assemble Cas9 RNPs.

1.3. Combine the following reagents to prepare injection mixes containing desired construct(s) to integrate.

· Your plasmid(s) of interest at desired concentration, typically for between 1-50 ng/µl in the final injection mix depending on desired expression level and promoter(s) used.

· An *unc-119* rescue plasmid (eg pCFJ108 Addgene #200367), 5 ng/µl final concentration. This can be omitted if an *unc-119* rescue cassette is included in the plasmid(s) of interest.

· *Optional* , an additional co-injection marker at desired concentration.

· Carrier DNA (eg Invitrogen 1 kb+ DNA ladder), to adjust total DNA concentration of the injection mix to 100 ng/µl

· Adjust volume with nuclease-free water to 13 µl

· Add 2 μl of tdTomato-targeting Cas9 RNPs prepared above (15 µl total volume).

1.4. Spin injection mix for 5 minutes at maximum speed on a benchtop microcentrifuge, transfer 13 µl of injection mix to a fresh tube to remove debris.

1.5. Store injection mixes at 4˚C. Mixes may be stored for up to ~6 months before use.

2. Injection and selection of integrated transgenes.

2.1. Inject Unc tdTomato landing pad animals (see Table 1).

· Note: *unc-119* animals should be raised at 15˚C or 20˚C prior to injection. Inject enough animals to ensure recovery of at least 10 that were successfully injected. Depending on experience of the injector and/or toxicity of the constructs, injection success rates can vary from 10-100%.

2.2. Place each injected P0 on a separate plate, incubate at 25˚C for 7-10 days.

· Note: it is beneficial to screen plates for moving worms ~3 days after injection to know how many injections were successful and whether more injections are needed.

2.3. After 7-10 days, after plates have exhausted the bacterial food source, identify plates that contain many non-Unc animals. From each plate that contains many (>100s) of non-Unc animals, transfer some animals to a fresh plate by chunking. Return plates to 25˚C.

2.4. The next day, single 8-10 non-Unc tdTomato(-) L2s/L3s from each chunked plate. Place singled animals at 25˚C.

· Note: On most plates, all moving animals are tdTomato(-). It can save time to initially screen plates to confirm that all animals are tdTomato(-) and then pick non-Unc animals without checking for red fluorescence.

2.5. After 3-4 days, screen plates to find animals that are homozygous for an integrated transgene as indicated by 100% non-Unc progeny.

· Note: Exclude plates with any number of Unc animals, even if very few. These animals harbor a very stable array, not a homozygous integrated transgene.

· Note: Propagate candidate integrants for 1-2 additional generations to confirm that no Unc animals are present.

· Note: Only keep one confirmed integrant derived from each injected worm. Even though a single injected worm may theoretically give rise to multiple independent integration events, these are not possible to distinguish.

· Note: confirm loss of tdTomato in integrated lines.

## Reagents

**Table:** 

**Strain #**	**Genotype**	**LG (cM)**
NJL3729	nicTi600[eft-3p::tdTomato::H2B unc-119(-) * oxTi556 ] I; unc-119( ed3 ) III	I (1.23)
NJL3730	nicTi601[eft-3p::tdTomato::H2B unc-119(-) *oxTi726] II; unc-119( ed3 ) III	II (-1.02)
NJL4308	nicTi605[eft-3p::tdTomato::H2B unc-119(-) *oxTi612] unc-119( ed3 ) III	III (-4.08)
NJL3878	unc-119( ed3 ) III: nicTi603[eft-3p::tdTomato::H2B unc-119(-) *oxTi705] IV	IV (0.09)
NJL4309	unc-119( ed3 ) III; nicTi606[eft-3p::tdTomato::H2B unc-119(-) *oxTi391] IV	IV (12.29)
NJL3731	unc-119( ed3 ) III; nicTi602[eft-3p::tdTomato::H2B unc-119(-) *oxTi392] V	V (-1.51)
NJL3879	unc-119( ed3 ) III; nicTi604[eft-3p::tdTomato::H2B unc-119(-) *oxTi400] X	X (1.43)

**Table 1. List of strains generated for FLInt with *unc-119* rescue.** All strains will be deposited and made available from the CGC.
